# Cingulate seizure-like activity reveals neuronal avalanche regulated by network excitability and thalamic inputs

**DOI:** 10.1186/1471-2202-15-3

**Published:** 2014-01-03

**Authors:** José Jiun-Shian Wu, Wei-Pang Chang, Hsi-Chien Shih, Chen-Tung Yen, Bai Chuang Shyu

**Affiliations:** 1 Department of Life Science, National Taiwan University, Taipei 10617, Taiwan; 2 Institute of Biomedical Sciences, Academia Sinica, Taipei 11529, Taiwan; 3 Graduate Institute of Life Sciences, National Defense Medical Center, Taipei 11490, Taiwan

**Keywords:** Neuronal avalanche, Epilepsy, Seizure-like activities, Thalamocortical circuit, Cortical dynamics

## Abstract

**Background:**

Cortical neurons display network-level dynamics with unique spatiotemporal patterns that construct the backbone of processing information signals and contribute to higher functions. Recent years have seen a wealth of research on the characteristics of neuronal networks that are sufficient conditions to activate or cease network functions. Local field potentials (LFPs) exhibit a scale-free and unique event size distribution (i.e., a neuronal avalanche) that has been proven in the cortex across species, including mice, rats, and humans, and may be used as an index of cortical excitability. In the present study, we induced seizure activity in the anterior cingulate cortex (ACC) with medial thalamic inputs and evaluated the impact of cortical excitability and thalamic inputs on network-level dynamics. We measured LFPs from multi-electrode recordings in mouse cortical slices and isoflurane-anesthetized rats.

**Results:**

The ACC activity exhibited a neuronal avalanche with regard to avalanche size distribution, and the slope of the power-law distribution of the neuronal avalanche reflected network excitability *in vitro* and *in vivo*. We found that the slope of the neuronal avalanche in seizure-like activity significantly correlated with cortical excitability induced by γ-aminobutyric acid system manipulation. The thalamic inputs desynchronized cingulate seizures and affected the level of cortical excitability, the modulation of which could be determined by the slope of the avalanche size.

**Conclusions:**

We propose that the neuronal avalanche may be a tool for analyzing cortical activity through LFPs to determine alterations in network dynamics.

## Background

The brain is a complex system, in which neurons can integrate various inputs and process certain functions [1,2]. Serious neuronal insult can break the excitation/inhibition (E/I) balance and cause severe dysfunctions [1-5]. Traditional clinical diagnoses focus on the electroencephalography (EEG) waveform and frequency. With the increase in computing power, the accurate and objective identification of collective cerebral activity transmission, including waves, oscillations, and synchrony, becomes more critical. The rules for signal-transferring processing generally include spatial and temporal correlations and coherence, and these rules are broadly used in the analysis of the EEG under spontaneous cortical activity in clinical examinations and animal studies. The spatial and temporal properties of these phenomena can be described by mathematical models, the neuronal avalanche, which was proposed by Plenz and Beggs, and could be a potential model to evaluate network dynamics [6]. The neuronal avalanche is a cascade of bursts of activity in neuronal networks, whose size distribution can be approximated by a power law distribution. Recent studies indicated that the neuronal avalanche could be found in both *in vitro* and *in vivo* recording systems [7-10].

Several complex systems, such as earthquakes [11,12] and forest fires [13-15], show similar activity transmission. A single unit with a specific threshold can dissipate activity back to the system as a processing cascade [15,16]. This dynamic has been found in neuronal networks, known as a *neuronal avalanche*, whose distribution possibility in local field potentials (LFPs) can be an approximation to a power-law distribution [6-8]. The neuronal avalanche is a cascade of bursts of activity in neuronal networks whose size distribution could be approximated by a power law distribution. Previous studies have proven the universality of this phenomena, and network activity could be optimized for information processing in this dynamic range, the critical state [7-10,17]. One of the main signatures in neuronal avalanche is the size distribution, which decays as a power-law with exponents, α, around −1.5 in local field potential and −2.1 in spikes. This slope, α value, may relate to the activities of the neuronal network. Recently studies indicated that the neuronal network behaved oscillation, which is proximal near the critical state. It is essential to maintain the E/I balance for homeostatic mechanism in the signal processing [18]. Now it is an important tool to monitor information processing in higher cortical function with a scale-invariant dynamics [19].

Some pathological conditions, such as seizures, may alter the cortical network and show a prolonged period of hyperactivity and an aberrant avalanche [20-22]. Simulations have indicated that the dynamic range of inputs is optimized in the critical state [6,7,17,23,24]. These studies, however, are still controversial with regard to the correlation of some parameters of the neuronal avalanche, such as the slope of the power-law distribution and the status of network activity. Furthermore, the cortical network displays spontaneous activity that persists in the absence of sensory stimuli. This is a robust feature of cortical dynamics because it is only modulated to a small extent by stimulus presentation [6,9,25]. However, the correlation between the inputs and neuronal avalanche is still unclear. Our previous study characterized the cortical neuronal avalanche in response to nociceptive stimulation *in vivo*[10,14]. The results implied that the slope of the power-law distribution in a network might be regulated by external inputs.

We previously demonstrated that seizure-like activity in the anterior cingulate cortex (ACC) could be induced by 4-aminopyridine (4AP) and bicuculline (Bic), antagonists of selected voltage-gated potassium channels and γ-aminobutyric acid-A (GABA_A_) receptors, respectively [11,13,26-28]. The ACC is mainly connected with thalamic nuclei whose inputs exert a desynchronous influence on epileptiform activity and inhibitory mechanism that further suppresses seizure augmentation [13]. We hypothesize that network excitability and thalamic input regulation can be revealed and quantified by calculating the slope of the distribution. The present study examined 4AP- and Bic-induced seizures with and without remote thalamic inputs. We hypothesized that the neuronal avalanche can be a tool to determine alterations in network dynamics *in vitro* and *in vivo*.

## Results

### Neuronal avalanche is evident in cortical seizure-like activity

**Figure 1:**
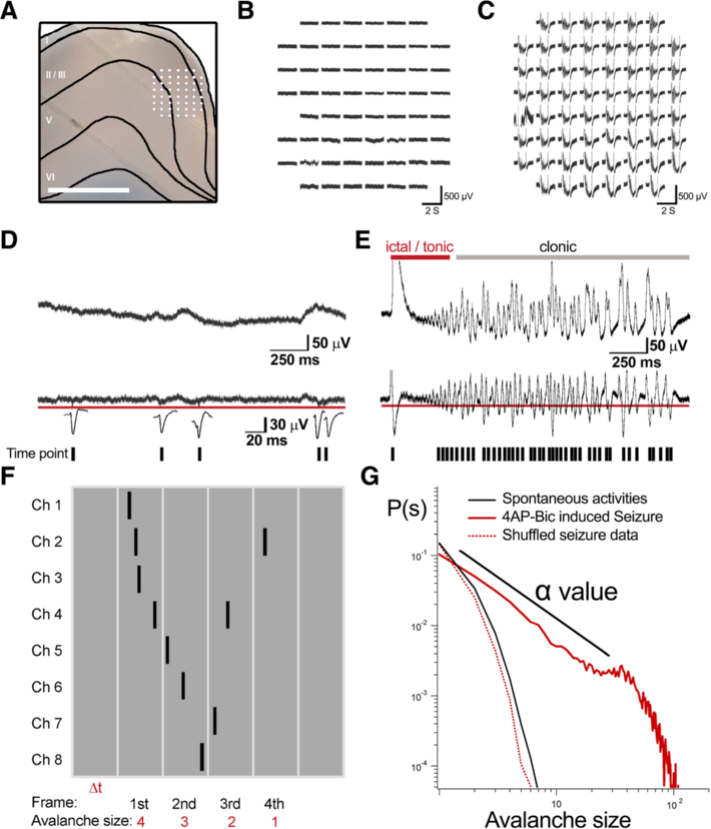
**Seizure-like activity and the definition of the neuronal avalanche. A**, Orientation of the brain slice to the recording site. **B**-**C**, Typical traces of spontaneous and seizure activity, which could be induced by applying 4AP and Bic. **D**-**E**, Seizure-like activity was composed of ictal events, followed by tonic and clonic bursts. The filtered nLFPs were set at threshold to detect neuronal activity and are shown in the upper and middle panels. The time-point selected from the nLFPs of each channel at which the nLFPs exceeded the specific threshold is marked as a digital unit in the lower panel. **F**, Example of the collective time step, which is framed in 4 ms time bins from eight channels. The definition of an avalanche is separated by blank activity at the beginning and end of the events. The activated electrodes are counted as the avalanche size, and each event’s lifetime is the summation of the total time frame. **G**, Distribution of different avalanche sizes plotted on a log-log scale. The neuronal avalanche could follow the power-law distribution, and its slope could be calculated as the α value. The original data were shuffled in their spatiotemporal arrangement to disturb intrinsic dependency. Both the spontaneous activity and shuffling data show a Poisson distribution. Scale bar = 1 mm in **A**.

The cingulate neuronal avalanche was examined in spontaneous and 4AP- and Bic-induced epileptic activity. The orientation of the brain slice and relative recording position of the multi-electrode array (MEA) are shown in Figure 1A. Significant seizure-like activity could be observed after the application of 4AP and Bic. Examples of the ensemble of activity traces before and after seizure induction are shown in Figure 1B and C, respectively. A threshold was set on the high-pass filtered sweep of negative LFPs (nLFPs) to detect the active neuronal responses, which are shown in the middle panels of Figure 1D and E. These nLFPs have been shown in previous studies to be correlated with neuronal spikes [6,27,29-32]. A small amplitude of action potentials developed in spontaneous activity as shown in the enlarged single trace in Figure 1D. Seizure-like activity in our previous study was composed of ictal/tonic bursts (Figure 1E, red line, upper panel), followed by clonic bursts (Figure 1E, gray line, upper panel; [13,21]. The time-point selected from the nLFPs of each channel at which the nLFPs exceeded the specific threshold is marked as a digital unit in the lower panels of Figure 1D and E. All of the time units could be pooled in a time-scale-framed plot to calculate the avalanche size and lifetime. Figure 1F shows a typical example of the collective time units that were framed by a time window (Δt, gray regions) from eight-channel recording. The avalanche is defined as a series of activity separated by a blank at the beginning and end of the events. The avalanche size was calculated as the total number of electrodes with active units, and the lifetime was calculated as the summation of the total time frame in each avalanche event. The distribution of the avalanche size with its probability *P(s)* were plotted on a log-log scale. A neuronal avalanche that has a fitted straight-line slope of α value indicates a power-law relationship and the event’s dependence. The 4AP-Bic group (red solid line) showed a power-law distribution with the α value around −1.5 (Figure 1G). To test whether event dependence is essential for the power-law relationship, the event dependence was disturbed, in which the original data were shuffled with regard to the order of both the temporal sequence and spatial arrangement of the events. Both the shuffling data (dashed red line) and spontaneous activity data (black line) showed an exponential distribution, which is a type of Poisson distribution in which the events occur independently (Figure 1G).

**Figure 2:**
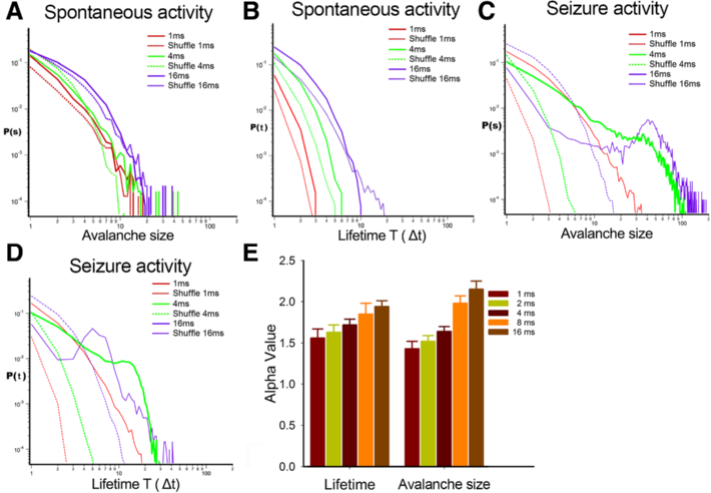
**Selection of the optimal time bin in the neuronal avalanche. A**-**B**, Both spontaneous activity and the shuffled group are fitted to an exponential distribution and have no power-law distribution of the avalanche size and lifetime in the different time bin groups. **C**-**D**, Different time bins in the seizure activity groups show a difference in the slope of the power-law distribution (*n* = 6, *F*_5,131_ = 28.167, *p* < 0.01, one-way ANOVA). **E**, The α value plotted in a bar chart. Different time windows are coded in different colors.

The avalanche size and lifetime of both spontaneous and induced seizure activity were plotted in different time bins. The distribution of the avalanche size and lifetime and each shuffled groups were fitted to an exponential distribution and had no power-law distribution in the different time bin groups (Figure 2A, B). The different time bins in the seizure activity groups showed a significant difference in the slope of the power-law distribution with regard to size and lifetime, respectively (Figure 2C-E; avalanche size, *n* = 6, *F*_5,36_ = 28.167, *p* < 0.01; lifetime, *F*_5,27_ = 12.274, *p* < 0.01; One-way analysis of variance, ANOVA). The 4 ms time bin was selected according to previous study [15,27]. The 4 ms time bin is an optimized selection of the neuronal avalanche when considering the speed of the spread of neuronal activity and distance of the nearest electrodes. These firing patterns are reproducible over periods of as long as 6 hours with the temporal precision of 4 ms. The seizure activity in the shuffled group was fitted to an exponential distribution and had no power-law distribution in the different time-bin groups.

### Neuronal avalanche in an enhanced network activity

**Figure 3:**
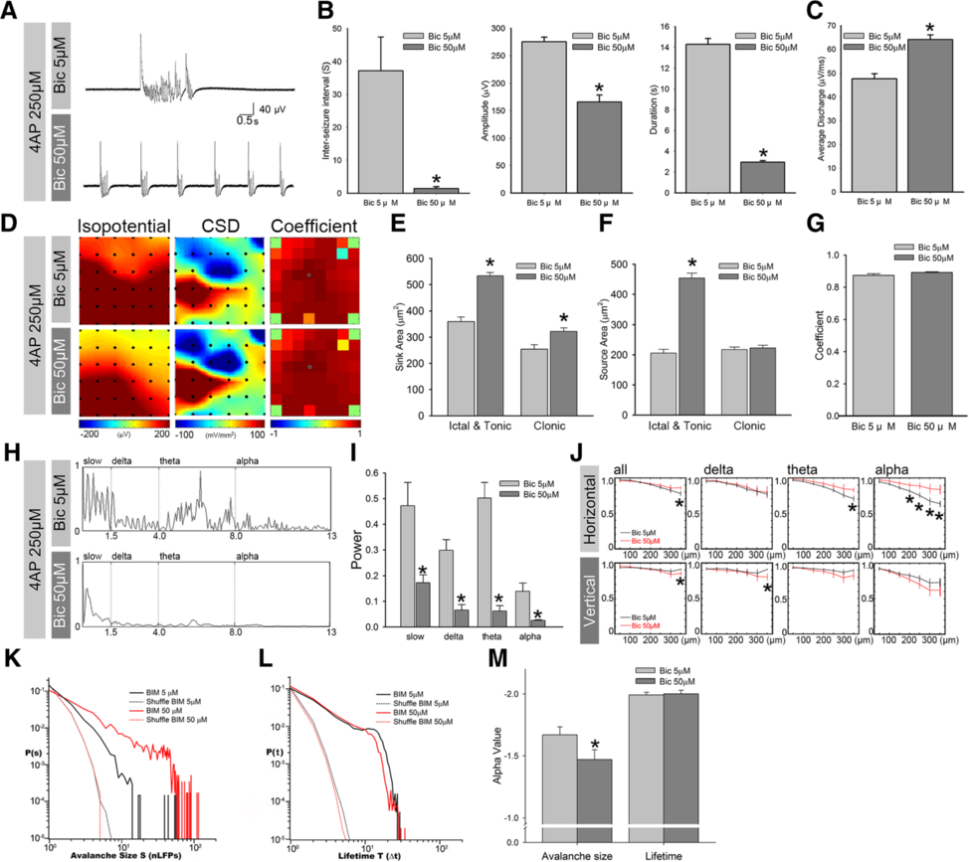
**Dynamics of the neuronal avalanche in enhanced network activity. A**, Typical traces of seizure-like activity with the application of low (5 μM) and high (50 μM) Bic concentrations. **B**-**C**, Comparison of amplitude, duration, average discharge, and inter-seizure interval (ISI) in a bar chart. **D**, Color map of isopotential, 2D-CSD, and correlation coefficient. **E**-**G**, 2D-CSD in the ictal/tonic and clonic phases of seizure activity. **H**-**I**, Power spectrum of FFT and statistical bar chart. **J**, Coherence of each frequency band in the vertical and horizontal directions. **K**-**M**, Avalanche size and lifetime distribution on a log-log scale. The shuffled groups showed a Poisson distribution (dash line). The statistical bar chart indicates that the avalanche size increased significantly after the application of the high concentration of Bic. *p < 0.01.

The excited state of induced seizure activity was positively related to the concentration of Bic [13,33]. Thus, the dynamics of the neuronal avalanche were tested with enhanced network activity induced by the application of a higher concentration of the GABA_A_ receptor antagonist Bic. A low concentration of Bic (5 μM) induced spontaneous seizure-like activity. A high concentration of Bic (50 μM) changed seizure-like activity into constant, short-duration bursts, with an inter-seizure interval of 2.28 ± 0.416 s (Figure 3A and B, left panel; Student’s *t*-test, *n* = 6, *p* < 0.001). The amplitude of seizure-like activity was significantly lower in the 50 μM Bic groups compared with the 5 μM Bic group (Figure 3B, middle panel; *n* = 6, *p* < 0.001). The duration of seizure-like activity was also significantly lower in the 50 μM Bic group (Figure 3B, right panel; *n* = 6, *p* < 0.01, Student’s *t*-test). The enhancement of network activity could be demonstrated in the average neuronal discharge measured in fixed 1 min recording periods, which significantly increased after the application of a higher dose of Bic (Figure 3C; *n* = 6, *p* < 0.001, Student’s *t*-test). The pseudo color of the isopotential plot showed that most of the brain regions covered by the MEA were involved in seizure-like activity (Figure 3D). To test whether the higher concentration influences network seizure-like activity, we used the 2D-CSD to analyze the distribution of neuronal activity. Both the sink and source currents were significantly increased after the application of 50 μM Bic, but the sink current was increased only in the clonic phase (Figure 3D-F; *n* = 6, *p* < 0.001). The cross-correlation coefficient showed no significantly difference between the 5 and 50 μM Bic treatment groups (Figure 3G).

The power density in each frequency band was significantly decreased in the slow-wave (0–1.5 Hz), delta (1.5-4 Hz), theta (4–8 Hz), and alpha (8–13 Hz) frequency domains in the 50 μM Bic group (Figure 3H and I; *n* = 6, *p* < 0.001). The brain slice was oriented so that the cortical layers were aligned with the horizontal channel direction. We calculated the coherence between the recording channels in cortical columns in the vertical direction or layer II/III in the horizontal direction. The coherence of each rhythmic band calculated in the horizontal and vertical directions was compared between the low-concentration (black line) and high-concentration (red line) Bic groups (Figure 3J). A high concentration of Bic significantly increased coherence in the horizontal direction in the alpha band compared with the low concentration group (Student’s *t*-test, *n* = 6, *p* < 0.01). In the vertical direction, coherence in the alpha band was significant decreased by the application of the high concentration of Bic (Figure 3J; Student’s *t*-test, *n* = 6, *p* < 0.01). This effect indicated that the potentiation of network activity may influence the propagation efficiency in different directions and within specific frequency bands [3,34,35]. The distribution of the avalanche size and lifetime distribution of the two states of network activity are shown in Figure 3K and L, respectively. Both of the network activities showed a power-law distribution, indicating that the neuronal network with drug-induced seizure activity exhibited scale-invariant dynamics (i.e., a neuronal avalanche). The α value was increased with the higher concentration of Bic from −1.67 to −1.47 (Figure 3M). However, the lifetime of the α value was not affected by the higher concentration of Bic (Figure 3L).

### Neuronal avalanche in a suppressed network

**Figure 4:**
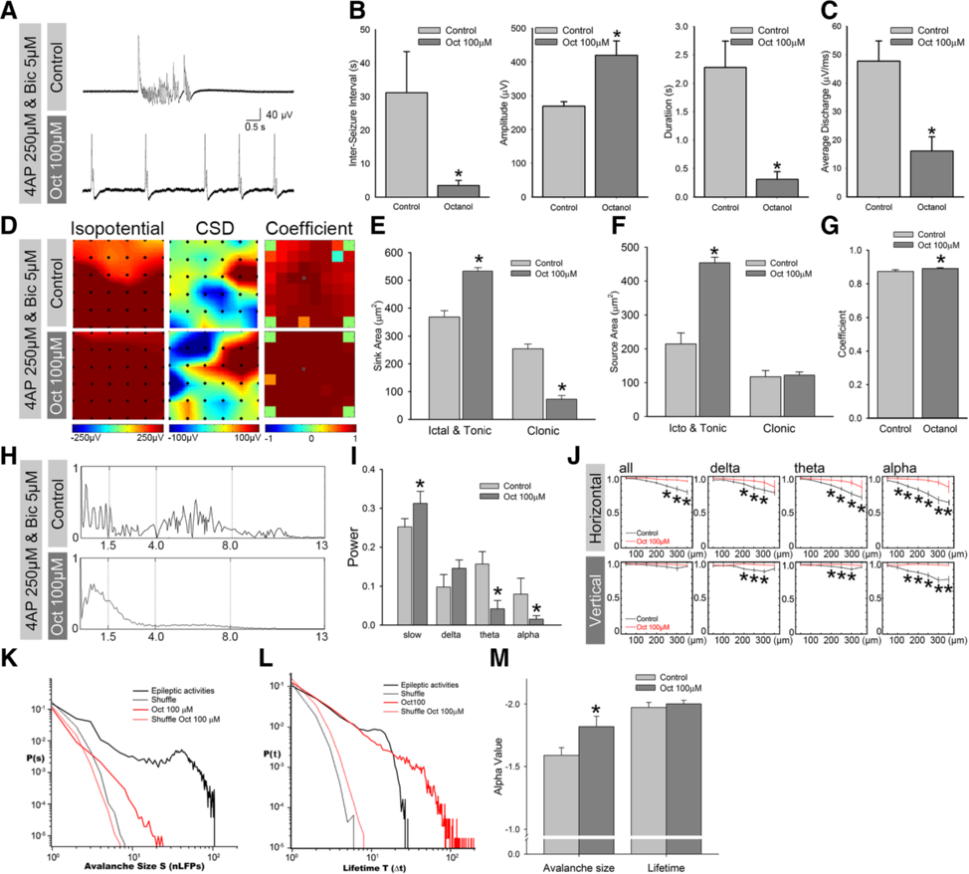
**Neuronal avalanche in a suppressed network. A**, Typical traces before and after application of the gap junction blocker Oct, which changed seizure-like activity into a constant, repeated, short-duration firing pattern. **B**-**C**, Comparison of ISI, amplitude, duration, and average discharge in a bar chart. Notice that the average discharge significantly decreased with a suppression of network dynamics. **D**, Color map of isopotential, 2D-CSD, and correlation coefficient before and after Oct application. **E**-**G**, 2D-CSD in the ictal/tonic and clonic phases of seizure activity and statistical bar chart. The application of Oct increased the correlation coefficient compared with the control group. **H**-**I**, Power spectrum of FFT and statistical bar chart. The power of the delta and alpha bands decreased in the Oct group. **J**, Coherence of each frequency band in the vertical and horizontal directions. The coherence in both the horizontal and vertical directions increased after Oct application. **K**-**M**, Avalanche size and lifetime distribution on a log-log scale. The statistical bar chart indicates that the avalanche size significantly decreased after the application of Oct. *p < 0.01.

Octanol (Oct), a gap junction blocker, has been used to reduce seizure-like activity [10,11,19,28]. We examined the avalanche size and neuronal avalanche lifetime in a suppressed neuronal network. Typical seizure-like activity and the effects of Oct are shown in Figure 4A. The application of Oct (100 μM) changed the seizure-like activity into constant, very short-duration bursts, with an inter-seizure interval of 3.14 ± 0.275 s (Figure 3B, left panel; Student’s *t*-test, *n* = 6, *p* < 0.001). Notice that the amplitude of each event was increased, and the duration of seizure activity was shortened after the application of 100 μM Oct (Figure 4B, *n* = 6, *p* < 0.001, Student’s *t*-test). Oct significantly decreased the averaged discharges measured in all recording traces and suppressed network activity (Figure 4C; *n* = 6, *p* < 0.001, Student’s *t*-test). To test whether Oct influences the synchronization of seizure-like activity, we analyzed the isopotential profile, two-dimensional current source density (2D-CSD) patterns, and cross-correlation coefficients. The 2D-CSD source and sink distribution areas were significantly increased in the Oct group in the ictal and tonic phases, but the sink distribution decreased only in the clonic phase (Figure 4D-F; *n* = 6, *p* < 0.001). The average cross-correlation coefficient was also significantly increased in the Oct treatment group (Figure 4G; Student’s *t*-test, *n* = 6, *p* < 0.01). The power of the theta and alpha bands was significantly decreased, and the slow wave increased after Oct administration (Figure 4H and I).

The coherence of each rhythmic band was calculated in the horizontal and vertical directions with the low Bic concentration (black line) and high Bic concentration (red line; Figure 4J). In the Oct group, coherence in the horizontal direction was significantly increased in the delta, theta, and alpha bands compared with the control group (Figure 4J, upper panel; Student’s *t*-test, *n* = 6, *p* < 0.01). In the vertical direction, coherence also increased in the same rhythm group (Figure 4J, lower panel; Student’s *t*-test, *n* = 6, *p* < 0.01). The distribution of the avalanche size and lifetime of two states of network activity are shown in Figure 4K, L, and M, respectively. The slope of the avalanche size was changed from −1.67 in the control group to −1.82 in the Oct group. No significant change in lifetime was observed. The α value of the avalanche size can be taken as an index of network dynamics. Based on the results presented in Figures 2 and 3, we found that the average discharge and α value of the avalanche size increased with enhanced network activity. The average discharges and α value were decreased in suppressed network activity.

### Cortical network dynamics regulated by medial thalamic inputs

**Figure 5:**
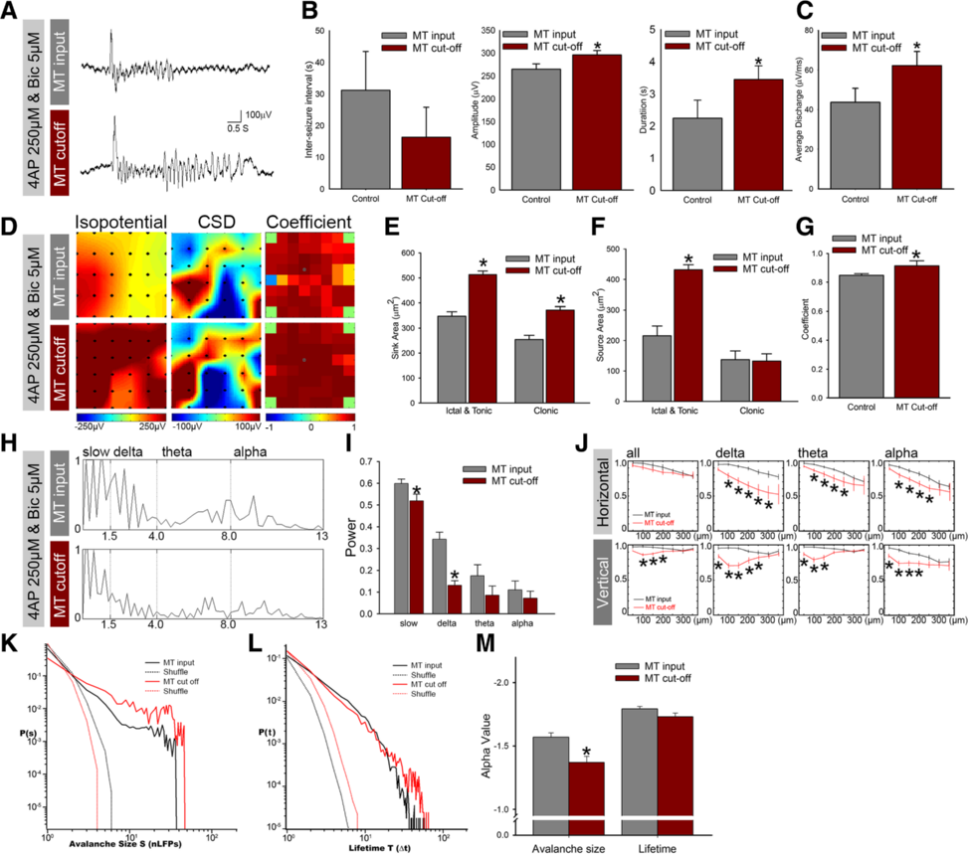
**Modulation of network dynamics by thalamic inputs *in vitro*. A**, Typical traces of seizure-like activity with and without medial thalamic inputs. The duration of epileptic activity was prolonged after cutting the thalamic inputs. **B**-**C**, Comparison of ISI, amplitude, duration, and average discharge in a bar chart. Notice that the average discharge significantly decreased after cutting the thalamic inputs. **D**, Color map of isopotential, 2D-CSD, and correlation coefficient with and without medial thalamic inputs. **E**-**G**, Bar chart of 2D-CSD in the ictal/tonic and clonic phases of seizure activity. Removal of the thalamic inputs increased the correlation coefficient compared with the control group. **H**-**I**, Power spectrum of FFT during seizure activity and statistical bar chart. The power of the low frequency and delta bands significantly decreased after cutting the thalamic inputs. **J**, Coherence of each frequency band in the vertical and horizontal directions. The coherence in the horizontal direction decreased more after cutting the thalamic inputs. **K**-**M**, Avalanche size and lifetime shown on a log-log scale. The statistical bar chart indicates that the avalanche size significantly increased in the group with cut thalamic inputs. *p < 0.01.

Our previous study showed that network activity in the ACC could be modulated by medial thalamic inputs in epileptic and nociceptive processing [13,15,18]. The present study investigated the effects of regulating thalamic inputs on the network dynamics of seizure-like activity in the ACC. Typical responses before and after blocking thalamic inputs are shown in Figure 5A. The inter-seizure interval did not significantly change after the interruption of MT inputs (Figure 5B, left panel). Both the duration and amplitude of epileptic activity were significantly increased after the removal of medial thalamic inputs (Figure 5B, middle and right panels; *n* = 6, *p* < 0.01, Student’s *t*-test). The effect of MT interruption significantly increased the average discharges measured in all recording traces and caused excitatory network activity (Figure 5C; *n* = 6, *p* < 0.001, Student’s *t*-test). To test the effect of cutting thalamic inputs on the influence of the synchronization of seizure-like activity, pseudo color isopotential plots, 2D-CSD patterns, and cross-correlation coefficients were generated and are shown in Figure 5D. The 2D-CSD analysis showed that the sink and source currents were significantly increased in the ictal and tonic phases, but the sink current increased only in the clonic phase after the removal of thalamic inputs (Figure 5E, F; *n* = 6, *p* < 0.001, Student’s *t*-test). The cross-correlation coefficient was also significantly increased compared with the intact thalamic input group (Figure 5G; *n* = 6, *p* < 0.05). The power spectrum pattern of rhythm bands decreased in the slow-wave and delta bands after cutting the thalamic inputs (Figure 5H, I; *n* = 6, *p* < 0.001). The coherence coefficient was significantly decreased in the delta, theta, and alpha bands in the horizontal direction after the thalamic inputs were cut (Figure 5J, upper panel; Student’s *t*-test, *n* = 6, *p* < 0.01). In the vertical direction, coherence in the group with severed thalamic inputs was significantly decreased and returned to original levels in distal electrodes in the delta, theta, and alpha frequency bands compared with the intact thalamic input group (Figure 5J, lower panel; Student’s *t*-test, *n* = 6, *p* < 0.01). The avalanche size was significantly decreased after the removal of thalamic inputs (Figure 5K, M; *n* = 6, *p* < 0.01, Student’s *t*-test). However, lifetime was not significantly altered (Figure 5L, M; *n* = 6). According to these results, thalamic inputs may exert tonic inhibition of the ACC and regulate the neuronal avalanche of cortical network dynamics.

### Neuronal avalanche regulated by thalamic inputs in vivo

**Figure 6:**
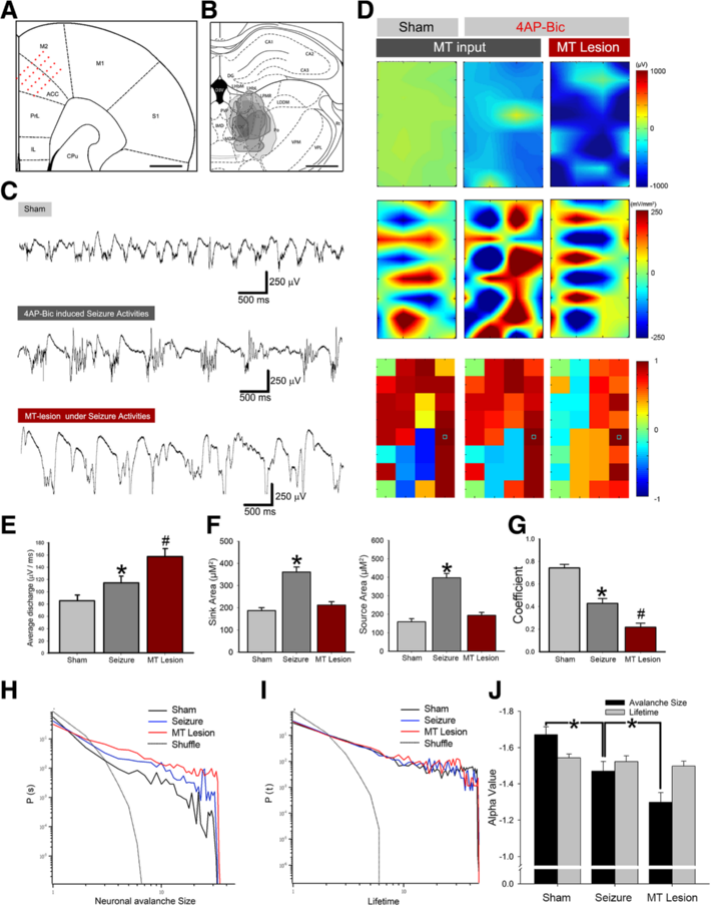
**Modulation of network dynamics by thalamic inputs *in vivo*. A**-**B**, Orientation of the recording site and areas of thalamic lesions plotted on an atlas. **C**, Typical field potential recording traces of sham group, induced seizures and after medial thalamic lesion. **D**, Color map of isopotential, 2D-CSD, and correlation coefficient. The reference electrode of the correlation coefficient is marked with a green rectangle. **E**, Comparison of average discharge. **F**-**G**, Bar chart of the 2D-CSD and correlation coefficient. **H**-**I**, Avalanche size and lifetime distribution shown in a solid line. **J**, The α value increased during induced seizure activity, and medial thalamic lesion increased the α value further. Scale bar = 1 mm in **A** and **B**. *p < 0.01.

Our previous studies indicated that the neuronal avalanche could be revealed in network activity that processes nociceptive information in vivo [15]. We used a 4 shanks with 8 channels of Michigan probe to record oscillation in the ACC and test whether MT inputs modulate oscillation in vivo. Figure 6A and B show the orientation of the electrodes and MT lesion area, respectively. Electrode tracks were labeled by 1,1-Dioctadecyl-3,3,3′,3′-tetramethyl-indocarbocyanine perchlorate (DiI) fluorescence. Typical traces of oscillation in the ACC are shown in Figure 6C. Spontaneous oscillatory activities in sham group turned into epileptic activity after the local surface application of 4AP (30 μM/50 μl) and Bic (5 μM/50 μl). Seizure-like activity was significantly changed after electrolytic lesion of the MT. The isopotential, 2D-CSD, and correlation coefficient of different network activities are shown in Figure 6D. The statistical results showed that the average discharge in seizure-like activity was significantly increased (Figure 6E; *n* = 6, *p* < 0.001, Student’s *t*-test). After MT lesion, the average discharges were further increased compared with the seizure group (Figure 6E; *n* = 6, *p* < 0.001, Student’s *t*-test). The sink and source current area in the 2D-CSD were significantly increased after seizure induction but reduced to control levels after cutting the thalamic inputs (Figure 6F; *n* = 6, *p* < 0.01, Student’s *t*-test). The correlation coefficient was decreased with seizure-like activity and decreased further after MT lesion compare with the seizure group (Figure 6G, *n* = 6, *p* < 0.01, Student’s *t*-test). The slope (α value) of the neuronal avalanche size significantly increased with seizure induction. The α value was further increased after MT lesion (Figure 6J; *n* = 6, *p* < 0.001, Student’s *t*-test). These *in vivo* results were correlated with the *in vitro* results shown in Figure 5. The thalamic inputs contributed to negative modulation with drug-induced seizure-like activity. The slope of the neuronal avalanche size distribution could reveal this modulation.

Several parameters are summarized in Table 1. The beta value was calculated by fitting the slope of the avalanche distribution after the cut-off tail. The beta value was significantly increase after the MT lesion in vitro (*n* = 6, *p* < 0.001, Student’s *t*-test) but not in vivo. Each neuronal avalanche displayed the power-law distribution with a cut-off tail. We calculated the avalanche size of each peak in the tail part of the power-law distribution as the peak point. The peak point changed from 22.8 to 13.4 after MT lesion in vitro (*n* = 6, *p* < 0.001, Student’s *t*-test) but not *in vivo*. The cut-off points that could be observed at the end of the power-law distribution with dramatic increases in the slope are summarized in Table 1. The cut-off point was increased significantly in the MT lesion group compared with the drug-induced seizure group in vitro (*n* = 6, *p* < 0.001, Student’s *t*-test). The branching parameter is an index of information processing distribution. The value of the branching parameter with the removal of thalamic inputs was 1.14 ± 0.24, which was not significantly different compared with the group with intact thalamic inputs in vitro and in vivo (*n* = 6, Student’s *t*-test).

**Table 1:** Analytical values of the power distribution of the neuronal avalanche

	***In vitro***			
	**Beta**	**Peak point**	**Cut-off point**	**Branching parameter**
Seizure-like activity	−10.032 ± 0.379	22.8 ± 1.584	19.8 ± 5.954	1.317 ± 0.022
High bic (50 μM)	−8.6152 ± 0.837	44.2 ± 4.732*	34.5 ± 7.445*	1.322 ± 0.035
Oct (100 μM)	−9.710 ± 0.729	11.2 ± 1.018*	11.5 ± 2.339*	1.297 ± 0.041
Thalamic cut-off	−13.22 ± 0.053*	13.4 ± 0.456*	61.4 ± 5.341*	1.358 ± 0.020
	***In vivo***			
	**Beta**	**Peak point**	**Cut-off point**	**Branching parameter**
Spontaneous	−9.112 ± 0.512	21.9 ± 3.401	32.4 ± 1.147	1.221 ± 0.072
Seizure-like activity	−10.015 ± 0.692	24.8 ± 2.921	32.7 ± 1.098	1.329 ± 0.061
Thalamic lesion	−10.862 ± 0.462	26.3 ± 1.866	33 ± 0.912	1.365 ± 0.095

The data are expressed as mean ± standard error. *p < 0.01.

### Neuronal avalanche size and lifetime distribution

**Figure 7:**
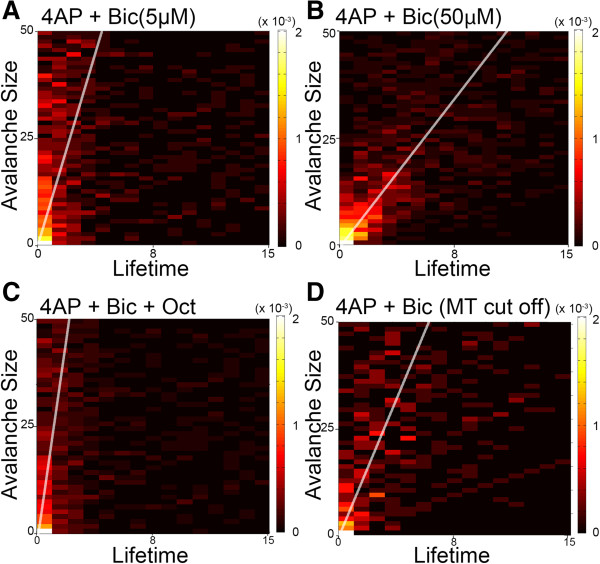
**Relationship between neuronal avalanche size and its lifetime. A**-**D**, Color map distribution of avalanche size and its lifetime. Lighter colors indicate a higher probability of total events in controls **(A)**, enhanced network activity **(B)**, suppressed network activity **(C)**, and thalamic input removal **(D)**. The white solid line represents the correlation of the avalanche size and lifetime of each group.

We found that the α value of the neuronal avalanche lifetime was not significantly changed by network dynamics with *in vitro* and *in vivo* recording. To evaluate the relationship between lifetime and size, the correlation and probability between the neuronal avalanche size and its lifetime were plotted on a color map (Figure 7). The white solid line represents the correlation regression line in each group. The high concentration of Bic exhibited a rightward shift in the correlation compared with the low concentration group. Oct treatment exerted an opposite influence compared with the control group (Figure 7A-C). The influence of cutting the thalamic inputs may be similar to the high-concentration Bic group and result in an enhanced network (Figure 7D). A positive correlation between lifetime and size was found in all of the experimental conditions. The cross-correlation coefficients of these two parameters, lifetime and size, were 0.031 ± 0.003, 0.055 ± 0.005, 0.018 ± 0.007, and 0.038 ± 0.011 in each group (*n* = 6, *F*_4,29_ = 0.023, *p* < 0.01, one-way ANOVA). The 50 μM Bic concentration decreased the value of the slope compared with the 5 μM group (*post hoc* test, *n* = 6, *p* < 0.01). The slope was significantly increased in the Oct group compared with the control group (*post hoc* test, *n* = 6, *p* < 0.01). The group in which thalamic inputs were cut showed no significant change compared with the 5 μM Bic group.

## Discussion

### Validation of the neuronal avalanche in *in vitro* and *in vivo* recording

The spontaneously activity of brain slices from adult mice did not exhibit a neuronal avalanche because they lacked synchronized activity. Excitatory neurotransmitters can induce more spontaneous activity and display a neuronal avalanche [7,8]. We tested the neuronal avalanche in drug-induced seizures *in vitro* and *in vivo*, in which robust ongoing activity generalized to lighter anesthesia. Previous studies have demonstrated the neuronal avalanche in these recording systems [10,17].

### Network dynamics in spontaneous cortical activity and neuronal avalanches

Spontaneous neuronal oscillations in cortical circuits have been described with regard to several aspects, such as the phase of activity, frequency coherence, and propagation patterns [20,22]. The distribution of the avalanche size with its probability could be roughly fitted by the power-law in a scale-free event size [6,7,17]. We found that the slope of the neuronal avalanche size was approximately −1.4 to −1.6 with *in vitro* and *in vivo* recording, which is within the range reported in previous studies [6,9]. We found a significant correlation between excitability and the α value. The α value would change within a range with the alteration of network activity, showing an inverted-U dose-dependent dopamine-NMDA regulation relationship [8]. Optimal stimulation and moderate activity might maximize the occurrence of oscillation and spatiotemporal correlations [26-28]. The inhibitory system, however, can shift network dynamics and impair the signal processing of epileptic activity, with the possible involvement of GABA_A_ receptors [27,29-32]. Previous studies found that disinhibition of GABA_A_ receptors altered cortical oscillations. We found that the slope of the neuronal avalanche was positively correlated with cortical excitability within a network of increased under GABA_A_ receptor blockade. The present results suggest that network modulation in an inhibitory system may be different from an excitatory system.

### Neuronal avalanche in seizure activity

A cortical seizure could be induced by abnormal excessive or synchronous neuronal activity. The relationship between the neuronal avalanche and seizure-like activity has been reported in a previous study. An aberrant neuronal avalanche was reported for cortical tissue that was removed from a juvenile epilepsy patient [21]. This indicates that neuronal avalanches are abnormally regulated in slices that are removed from epilepsy patients. This tissue exhibited prolonged periods of hyperactivity and an increase in the branching parameter. Our study experimentally demonstrated that the α value correlated with total activity *in vitro* and *in vivo*. To avoid pathological seizures, cortical networks maintain moderate average synchrony with maximally variable synchrony [27]. These results suggest that the distinctions between health and disease are scale-dependent. What is abnormal and the definition of dysfunction are not the propagation itself but rather activities that are sufficiently large to interfere with the normal function of the cortical network [33]. Our results indicate that the network excitability in certain seizure activities could be dramatically changed by the disinhibition of cortical activity and cause cortical dysfunction.

Our results strongly suggest that avalanche size is a more reliable indicator of network excitability than lifetime. We found that the lifetime remained unchanged in both enhanced and suppressed network activity. The correlation between size and lifetime showed a tendency toward an increase in slope in enhanced network activity, whereas the slope decreased in suppressed network activity. The change in slope could be explained by the decrease in the alpha value of size in enhanced network activity and increase in the alpha value of size in suppressed network activity. A previous study also reported that the scale-invariance in the avalanche size is accompanied by scale-invariance in the avalanche lifetime [9]. Our data showed that the lifetime distribution was scale-invariant and varied greatly, even for avalanches of any size, in which the large avalanche size tended to have a longer lifetime under more excitable network conditions.

The seizures are defined as an underlying transient abnormality of cortical neuronal activity in the clinical manifestation [36]. The phenotypic expression in seizure activities could be determined and characterized by its origin and the spreading in the spatial dimension, and the subsequent development and kindling progression in the temporal dimension [37]. In the spatial dimension, we demonstrate the neuronal avalanche could be detected and in limited cortical area, ACC, and it might be applied in the different and larger cortical area by the scale free manner [28]. In the temporal dimension, the seizure events usually consisted of ital., tonic and clonic phases and the underlying mechanisms of each individual stage are different [38,39]. In the present study, the neuronal avalanche describes the properties of the network activity of the whole series of seizure event instead of the individual stages of the events. In considering to calculate the avalanche of individual seizure stages, the duration of each individual seizure stage is significant shorter and cause the limitation to analysis the difference between the ictal, tonic and clonic state in the seizure activities. To collect sufficient data which covering the series of events from short duration to long duration, it will require the increase of the sampling time and sampling space. In the increase of the sampling space, it means that the number of electrodes in a multi- electrode array must be increased to record sufficient amount of the data. The electrode array we used in the present study only has 60 recording points and thus it is limited in sampling sufficient data for further analysis of the avalanche of individual seizure stage. Recently, a high-density multi-electrode array, CMOS-MEA, has been applied in neuronal recording [40]. Thus it is anticipated that the avalanche property of individual seizure stage could be resolved by using such high-density electrode array to gain the sufficient cortical seizure events in the limited temporal duration.

### Neuronal avalanche and EEG

Several aspects of the parameters analyzed in the present study deserve particular attention. In traditional EEG, the traces patterns, frequency distributions, and correlations between remote regions are important indices for evaluating cortical conditions [34,35]. The recurrence rate and 2D-CSD can measure the cortical neuronal state, which may represent an index of physiological homeostasis. However, the present results revealed some discrepancies, in which these parameters may not faithfully represent network excitability. For example, the amplitude and duration of typical activity and alterations in the 2D-CSD areas were not correlated with the excitability of network activity. Previous studies indicated that the neuronal avalanche could exhibited in cortical networks and might be potential candidates to measure brain activity in the processing of different tasks [10,19,28]. In the present study, we found that the slope of the power-law distribution could be a sufficient signature of cortical network excitability and contribute to the formation of criticality in the cortical network. Multi-level criticality may contribute to the subsequent class of dynamic systems, and each of them allows criticality to jointly emerge at multiple levels separated by a characteristic scale, which is traditionally considered contradictory in systems with self-organized criticality [18]. Scale-free dynamics of oscillatory neuronal networks would provide important insights into clinical diagnosis.

### Local cortical activity could be modulated by thalamic inputs

In this study, remote thalamic inputs could modulate cortical signal processing as a negative input to 4AP- and Bic-induced cortical seizures, and this modulation could be determined by the α value of the avalanche size *in vitro* and *in vivo*. Thalamic relay neurons synapse onto both excitatory and inhibitory neurons in cortical regions. The synapses between the thalamus and inhibitory interneurons are much stronger than those between the thalamus and excitatory pyramidal neurons [41]. Thus, the thalamic inputs could restrain the firing of pyramidal neurons by disynaptic feedforward inhibition. We found that lesions of the thalamus enhanced cortical seizures, indicating that thalamic inputs might influence seizures through feedforward inhibition. Previous *in vivo* studies also showed that thalamic inputs might be involved in the termination of seizures [42]. The basal ganglia may act as an online control system to desynchronize thalamocortical activity and contribute to seizure termination [42]. On the other hand, previous studies indicated that medial thalamic inputs can regulate nociceptive processing in the cingulate cortex [1,2,15]. Peripheral noxious inputs may alter network activity in which the neuronal avalanche can reflect alterations in excitability. Medial thalamic inputs might also play a modulatory role in drug-induced cingulate cortical seizures, and the removal of this input may represent enhanced network dynamics [1,2,4,5,13]. In the present study, we demonstrated that epilepsy could be modulated by external inputs and alter network activity with the confinement of spatiotemporal scales of these power-law phenomena. Some studies indicated that epilepsy results from a failure of modulation, possibly located in part of the cortex itself or in deep brain nuclei [12,43]. Furthermore, some studies indicated that network stability can be maintained and well-tuned by homeostatic plasticity via remote inputs, which might be crucial in critical-state organization and cortical function [8,14,18,44].

### Network dynamics and excitation/inhibition balance

The traditional evaluation of cortical seizures is based on analyzing the spatiotemporal distributions of EEG signals under physiological and pathological conditions. Previous studies indicated that self-organized criticality that occurs over a limited range of E/I conditions contributes to neuronal avalanches and peak information capacity and emerges together with balanced E/I [10,16,27,45,46]. In this study, we used Oct, which is known to act on T-type calcium channels to suppress network activity [47]. However, previous studies showed that T-type calcium subunit (α1G^−/−^) knockout mice exhibited normal susceptibility to 4-AP-induced tonic-clonic seizures [48], suggesting that T-type calcium channels are not involved in the pathogenesis of 4-AP-induced seizures. The convulsant we used in this experiment was 4-AP, which is a potassium channel blocker that affects A-type and D-type K^+^ currents [49,50]. The epileptogenetic mechanism of 4-AP administration might be attributable to the enhancement of both excitatory and inhibitory transmission [51] and depolarization of the membrane potential. Several studies showed that the application of ethosuximide, a T-type channel blocker, did not suppress 4-AP-induced seizure activity *in vivo* or *in vitro*[52,53]. Therefore, we concluded that the major effect of Oct in suppressing 4-AP-induced seizure occurred through the regulation of gap junctions.

The neuronal avalanche reveals the constitution of scale-invariant cortical synchronization in three principle dimensions: temporal sequence, spatial distribution, and clustered neuronal activity [6,9]. These principle properties may represent network dynamics to calculate synchrony and dispersion, which are manipulated by the network E/I balance. These mechanisms are dysfunctional in several type of seizure disorders and cause changes in the E/I balance of cortical networks [4,7-10]. Furthermore, the tuning of the activities in brain networks is essential for the criticality on multiple levels of neuronal organization, in which the power-law scaling can emerge on multiple temporal scales in constitutive oscillating networks [18]. The slope of the distribution in the lifetime of the neuronal avalanche is not significantly changed and represents the general properties of cortical networks. Thus, the slope of the avalanche size might provide a range of tuning of network activity. The increase of the alpha value could represent the more excitable status of the neuronal network activities in the physiological and pathological condition and vice versa.

### Functional application of the neuronal avalanche

Several studies that applied the neuronal avalanche using EEG have found that avalanche dynamics are related to long-range temporal correlations [21,54-56]. The repertoire of neural activity patterns may constrain and maximize the ability of the network to transfer and process information [23,24,26,27]. The present results may provide insights into the evaluation of information processing and dynamic alterations between physiological and pathological conditions [25,46,57-59]. Future investigations of physiological functions and pathological conditions in macroscopic scale networks should be conducted.

## Conclusions

In the present study, we emphasized the slope of the neuronal avalanche and comparisons with traditional aspects of LFP analysis. Power-law behaviors in cortical activity were associated with oscillations and could potentially be reflected in excitability and relayed inputs. Anterior cingulate cortex activity exhibited a neuronal avalanche. The slope of the avalanche size was sensitive to the change in network excitability and may reveal insights into higher functions in the cortex. Thus, the slope of the avalanche size could be a useful index to indicate network dynamics.

## Methods

### Animals

In brain slice recordings, we used 4- to 6-week-old mice (25–35 g body weight, C57Bl/6 J) that were housed in groups of five per cage with a 12 h/12 h light/dark cycle at 22°C. To maintain a similar recording site and avoid serious damage in cortical areas caused by the four shanks of the Michigan probe, male Sprague–Dawley rats (300–400 g) were used for the in vivo experiments. The mice and rats were housed in an air-conditioned room with free access to food and water. All of the experiments were performed in accordance with the guidelines established by the Academia Sinica Institutional Animal Care and Utilization Committee. Efforts were made to minimize animal suffering and reduce the number of animals used.

### Acute slice preparation

The mouse brains were removed from halothane-anesthetized animals and cooled for 3 min in chilled, oxygenated artificial cerebrospinal fluid (aCSF; 124 mM NaCl, 4.4 mM KCl, 1 mM NaH_2_PO_4_, 2 mM MgSO_4_, 2 mM CaCl_2_, 25 mM NaHCO_3_, and 10 mM D-glucose, bubbled with 95% O_2_ and 5% CO_2_). Brain slices were prepared according to a previously published procedure [15,60]. Briefly, the thalamocingulate block was hand-cut with two sagittal cuts and two angled cuts that were ventral and parallel to the pathway. The brain block was attached to an angular plate with cyanoacrylate adhesive, and a cut was made just above the turning point of the pathway. The stage was unfolded, flattened, and glued onto the chamber stage of a Vibratome (Series 1000, Vibratome, St. Louis, MO, USA). The brain slices were cut in ice-cold oxygenated aCSF with a 400–600 μm thickness. The slices were incubated in oxygenated aCSF at room temperature for at least 1 h before recording. Brain slices prepared using this procedure can preserve the connectivity between the MT and ACC. The oblique section angle was aligned with the trajectory of the thalamic inputs, which could maintain the intactness of the columnar structure of the cingulate cortex.

### Recording *in vitro*

After 1 h preincubation in oxygenated aCSF at room temperature, each slice was transferred to a 60 channels MEA probe (Multi Channel Systems, Reutlingen, Germany). To cover the ACC, an 8 × 8 MEA with 100 μm electrode spacing was used in the experiment. The slice was positioned above the recording area on the MEA probe, and the upper region was aligned with one site of the ACC. A silver weight was placed on a net above the slice to provide mechanical stabilization. The chamber was kept at 30°C under continuous perfusion (2 ml/min) of oxygenated aCSF. Local field potentials were simultaneously recorded from 60 electrodes with high spatial and temporal resolution (inter-recording leads, 200 μm; total covered area, ∼1400 μm × 1400 μm). Local field potentials at each electrode were recorded against the bath electrode. 4AP (250 μM final concentration) and Bic (5 and 50 μM final concentrations) in aCSF were applied in the perfusion system to induce seizure activity. A 60-channel amplifier was used with a band-pass filter set between 1 Hz and 3 kHz (MEA-1060-BC, Multi Channel Systems, Reutlingen, Germany). The data were acquired using MC Rack software (Multi Channel Systems, Reutlingen, Germany) with continuous recording at a sampling rate of 10 kHz.

### Recording *in vivo*

Anesthesia was induced with 4% isoflurane in pure O_2_ in a semitransparent acrylic box. The animal’s head was fixed in a small-animal stereotaxic instrument (David Kopf Instruments, Tujunga, CA, USA) and maintained under anesthesia with 2% isoflurane during surgery. The Michigan probe (NeuroNexus, Michigan, USA) with 32 contact points (150 μm lead interval, eight leads on one shank, and four parallel shanks), was used to record extracellular field potentials in the right ACC. The DiI (Invitrogen Molecular Probes, Oregon, USA) was dissolved in isopropanol at a saturated concentration and coated on the Michigan probe three times to ensure successful coating. The animals were subsequently maintained under anesthesia with 1.25% isoflurane during the experimental session. The depth of anesthesia was continuously monitored using an anesthesia monitor (Capnomac Ultima, General Electric Company, Fairfield, CT, USA). 4AP (30 μM final concentration) and Bic (2 μM final concentration) were added to the aCSF (50 μl) and then directly applied on the surface of the ACC for seizure induction. A tungsten electrode was inserted into the MT (−3.2 mm from bregma; lateral, 0.8 mm; depth, 5.0 mm) to make an electrolytic lesion, which was performed with a 100 μA direct current for 100 s by a constant current pulse generator (Model 2100, A-M Systems, Carlsborg, WA, USA) to deactivate the MT. After the lesion, the animals were transcardially perfused with normal saline followed by 4% paraformaldehyde. The entire brain was gently removed, post-fixed in 4% paraformaldehyde for 24 h, immersed in 30% sucrose, and cryosectioned at a thickness of 50 μm. The brain sections were processed for Nissl staining to histologically confirm the recording and lesion sites.

### Data processing i*n vitro* and *in vivo*

The recording data *in vitro* and *in vivo* were directly digitized at 20 kHz without filtering. The data were acquired and transformed using MC Rack and MC Data Tool software (Multi Channel Systems, Reutlingen, Germany). The data were analyzed using a custom MATLAB program (MathWorks, Natick, MA, USA). Briefly, event activity was first evaluated and filtered with a 200 Hz high-cut. To detect oscillatory events, we set two to four standard deviations (SDs) of the noise level as the threshold which is according to each background noise. The amplitudes of the peaks during an oscillation event and the seizure activities that surpassed this threshold were automatically detected by MC RACK software.

### General analysis of seizure activities

The analysis of the occurrence of seizure onset confirmed our earlier observations. Seizure-like activity induced by 4-AP and Bic were divided into ictal onset, a tonic phase, and a clonic phase based on frequency evolution shown by wavelets transformed from field potential recordings [61]. The frequency of oscillation was 6.5-10 Hz in the tonic phase and 2.5-4 Hz in the clonic phase. The duration of an oscillation event was measured by subtracting the time-point between the first and last peaks that surpassed the threshold. Epileptiform activity appeared in 10 ~ 15 minute after drug application. Our previous time control experiment indicated that the maximal and stable responses appeared between 2 to 3 hour *in vitro* and 1 to 2 hour *in vivo* after drug application [62]. Seizure-like activity was significantly reduced 4 hour *in vitro* and 3 hour *in vivo* after drug application. Thus we carefully design the experiment *in vitro* and *in vivo* during this period to exclude the concerning of the drug concentration changing and the immediately lesion effect in temporal scale. The color maps of the isopotential and 2D-CSD were calculated and constructed from the ictal peaks of field potential profiles [11,13]. Blue represents current sinks, and red represents current sources in 2D-CSD color map profiles. The boundary site data were obtained by extrapolation. Correlation coefficients have been used to measure the degree of synchronization of two coupled neurons [6,63]. The correlation coefficient is a free and scale-invariant parameter for measuring the degree of synchronization quantitatively. The correlation coefficient is a free and scale-invariant parameter for measuring the degree of synchronization quantitatively. The correlation coefficient is a free and scale-invariant parameter for quantitatively measuring the degree of synchronization. Briefly, one specific channel where the first ictal event of each epileptic oscillation was initiated in layer II/III of the ACC was selected as the calculation reference. The correlation coefficient color map was constructed from this selected channel and the remainder of the channels [13]. The coherence coefficients by the same reference channel was calculated from the cross-spectral density [64,65]. The power spectrum of the frequency distribution was collected from a typical response and calculated by Fast-Fourier Transformation (FFT). The intensities in power were normalized to values between 0 and 1. The coherence analysis was calculated from collected LFPs from the electrodes along the horizontal and vertical direction aligned with layer II/III and the cortical column, respectively, and analyzed using custom MATLAB programs.

### Neuronal avalanche analysis

The nLFPs of seizure-like activity were used to calculate the distribution of the avalanche size and its lifetime. The time-point in nLFPs of each channel was detected from filtered data that reached 2 SDs for the in vitro recording and 4 SDs for the in vivo recording. This time-point was marked as the digital unit for further neuronal avalanche calculation. The processed data thus contained a serial time-point of nLFPs and could be framed by selected time bins. The 4 ms time bin was the optimal selection for assessing the neuronal avalanche when considering the speed of the spread of neuronal activity and distance of the nearest electrodes (200 μm). We calculated the average inter-event interval, which is defined as the interval between LFPs that occurred at all electrodes, to ensure that each of the counted events were successive from other electrodes. The LFP data were binned at finer temporal resolution, making it clear that LFPs did not appear at all electrodes at exactly the same time. Each avalanche size is defined as the summation of the number of digitized units in a single avalanche event. The framed numbers of various neuronal avalanche sizes were counted for the avalanche lifetime distribution. The avalanche size and lifetime distribution were calculated using custom MATLAB software and plotted using SigmaPlot software (Systat Software, Chicago, IL, USA). The slope of the power-law distribution was calculated by fitting the front of the power-law distribution (α value) and cut-off tail (β value) using the MATLAB program. The cut-off point and sudden bump point before the cut-off were collected and calculated. These recording data were further shuffled to disturb the spatial and temporal dependence to verify the spatiotemporal dependency of the neuronal avalanche in the power-law distribution. The exponential fitting was also performed, and only R square values > 0.9 were included in the subsequent analysis.

### Branching parameter analysis

The branching parameter σ was calculated to describe activity propagation, the method of which was based on the studies by Beggs and Plenz [6,7,13]. The σ is defined as the average number of descendent-activated electrodes, which is the origin from one ancestor electrode [15,66]. Each avalanche event could be separated into several steps, and each step has descendant and ancestor electrodes.

(1) $$\sigma =\sum_{d=0}^{n_{\max}}d\times p\left(d\right),$$

The branching parameter σ was given by 1 in the case of only one ancestor, in which *d* is the number of the descendants, *p(d)* is the probability of the activated descendants, and *n*_*max*_ is the maximal number of active electrodes in each event.

### Statistical analysis

The statistical analyses were performed using SPSS software (SPSS, Chicago, IL, U.S.A.). The data are expressed as mean ± standard error, and *n* indicates the number of slices or animals studied. The ANOVA and Tukey’s *post hoc* test were used to analyze group differences in the correlation of the avalanche size and lifetime. Student’s *t*-test was used to analyze the effects of network excitability and the medial thalamic inputs in general and perform the avalanche analysis. The results were considered significant at *p* ≤ 0.05.

## Competing interests

The authors declare that they have no competing interests.

## Authors’ contributions

JJSW, WPC and HCS participated in the design of the study, conducted the experiments, analyzed the data, and drafted the manuscript. CTY participated in the discussion of the experimental results and made experimental suggestions. BCS conceived of the study, participated in its design and coordination, and participated in the writing of the manuscript. All of the authors read and approved the final manuscript.
